# Dataset on energy consumption in ore-processing plants in Zaruma-Portovelo, Ecuador

**DOI:** 10.1016/j.dib.2020.106019

**Published:** 2020-07-13

**Authors:** Karl-Heinz Gaudry, Daniel Charro, Abdul Shaban

**Affiliations:** a Instituto de Investigacion Geológico y Energético (IIGE), *Av*. De la República E7-263 y Diego de Almagro, Edificio Sky, Quito, Ecuador; b Centre International de Formation Européenne (CIFE), 81 rue de France, F-060000 Nice, France; c School of Development Studies, Tata Institute of Social Sciences, Mumbai, India

**Keywords:** Energy consumption, Ore-processing plant, Comminution

## Abstract

Based on 74 ore-processing plants and their energy meter's coordinates in Zaruma-Portovelo, we determined the energy consumption profiles for each ore-processing plant following the methodologies of Gonçalves (2016) and Cabezas (2016). In doing so, the total energy consumption in comminution processes was calculated. Data show a rising trend in energy consumption in ore processing in Zaruma-Portovelo. The data can be of immense use in designing sectoral reforms for transparency in the mining sector and developing cross-sectoral coordination between ARCOM and ARCONEL. The data can also help the state agencies in assessing the quantity of ore processed and preventing the leakage of revenue from the extractive sectors (Gaudry et al., 2020). Finally, the scientific community may benefit from this data, particularly those working on energy consumption, efficiency, and its forecasts in extractive sectors (Gaudry et al., 2020).

Specifications Table

**Table:** 

Subject	Energy
Specific subject area	Energy consumption in ore comminution processes
Type of data	Table
How data were acquired	SEM Instruments: Web-based geoportal, Excel
Data format	Raw Analysed
Parameters for data collection	The data went through several identifications, processing, and time measurement conditions. We first identified all the processing plants (with identification numbers) active in Zaruma-Portovelo. Out of these plants, we identified those under “industrial” categories of energy tariffs. The energy consumption [kWh] and number [NUM] of plants were accounted for within this region for the period 2010–2018.
Description of data collection	From a technical report published in 2014 by the Ecuadorian National Institute of Geological Mining and Metallurgical Research (INIGEMM), we identified an initial sample of 65 potential ore-processing plants in Zaruma-Portovelo. The names of the companies in initial sample were verified with names available with the National Electricity Corporation's geoportal in which we associated ore-processing location (coordinates) with those energy meters under the layer and attributes table “CNELEP - Conexión Consumido” (See: https://geoportal.cnelep.gob.ec/cnel/). Once verified, we expanded the sample of potential ore-processing plants by including additional inferred ore-processing plants within the exclusion zone of Zaruma. Based on the resulting 185 potential ore-processing plants, we requested the monthly energy consumption between 2008 and 2019 from ARCONEL, as well as their electricity tariffs. But, we were provided data until 2010–2018. The next, we obtained the monthly electricity consumption as well as the associated electricity tariff and limited the original sample of potential ore-processing plants to 74 (from 185) by filtering those with an industrial electricity tariff only. See: Total processing plants Based on such identified 74 ore-processing plants and their energy meters’ coordinates, we requested the following data from ARCOM: a) ore-processing tonnage declaration, b) type of mineral benefit process, and c) monthly data on refining and marketing of final products during 2008–2019. See: Total energy consumption (kWh) Having obtained the above-mentioned data we proceeded it to determine the energy consumption profiles for each ore-processing plant following the methodologies proposed by Cabezas et al. [1], Comisión Chilena del Cobre (2014), and Gonçalves [4] (See: Energy consumption in comminution (kWh))
Data source location	Empresa Eléctrica Pública Estratégica Corporación Nacional de Electricidad (CNEL-EP), https://geoportal.cnelep.gob.ec/cnel/ Agencia de Regulación y Control de Electricidad (ARCONEL), Avenida Naciones Unidas 7–71, Quito 170,506, Ecuador
Data accessibility	Energy meters and their coordinates are hosted under the public enterprise CNEL-EP geoportal: https://geoportal.cnelep.gob.ec/cnel/, specifically under the layer and attributes table. Further, data is hosted within the article [3] or upon request at ARCONEL. Instructions for accessing these data: Access CNEL-EP geoportal, identify energy meters, acquire energy meter code, request ARCOM energy consumption by energy meter code.
Related research article	Co-submission: Gaudry K.H., Charro D.M., Villacreses G., W. Castillo, A. Shaban [3]. Estimating Scales of Gold Ore Production and Processing in Zaruma and La Merced de Buenos Aires, Ecuador: electrical power consumption as an indirect metric. The Extractive Industries and Society, In Press.

Value of the Data

• The data show a rising trend of energy consumption in ore processing industries in Zaruma-Portovelo.
• The data can be used to understand the issues related to ore-processing industries and design new sectoral reforms for promoting transparency, and cross-sectorial coordination between ARCOM and ARCONEL.
• The data show significant positive correlation between ore processing and electricity consumption (billing). The electricity consumption data can be used by the state departments for estimating the actual quantity of ore processed by each industry and as such capturing the non-realised share of revenue.
• The scientific community may benefit from this data, particularly those working on energy consumption, energy use efficiency, and planning and development of extractive sector.
• The data can serve as baseline or point of reference to be used in future for evaluations of mining sector, performance of processing plants, official and estimated data (based on electricity consumption) on ore-processed.

## Data description

Table 1: Energy consumption in comminution processes by plants performing under "industrial artisanal" and "industrial hourly demand" energy tariffs (2010–2018). Data columns describe the ore-processing plants number ID and their coordinates in [WGS 84 S17]. Each ore-processing plant is described in terms of its yearly energy consumption. Some ore-processing plants have no data, indicating that they were inactive or non-present during the 2010–2018 period. Total processing plants (per year), total energy consumption (kWh) per year and energy consumption in comminution (kWh) per year are presented in the last row.

## Experimental design, materials and methods

To compute the energy consumption of ore-processing plants, we first identified the total number of potential ore-processing plants and obtained their energy meter code and thus their energy consumption. Using the technical report published in 2014 by the Ecuadorian National Institute of Geological Mining and Metallurgical Research (INIGEMM), we identified an initial sample of 65 potential ore-processing plants in Zaruma-Portovelo. The initial sample was verified at the National Electricity Corporation's geoportal in which we associated ore-processing location (coordinates) with those energy meters under the layer and attributes table “CNELEP - Conexión Consumido” (See: https://geoportal.cnelep.gob.ec/cnel/).

Once verified, we expanded the sample of potential ore-processing plants by including additional ore-processing plants within the exclusion zone of Zaruma. Based on the resulting 185 potential ore-processing plants, we requested the monthly energy consumption during 2008–2019 from ARCONEL, as well as their electricity tariffs. However, we received the data for the period 2010–2018. Next, we limited the original sample of potential ore-processing plants to 74 (from 185) by filtering in those with an industrial electricity tariff only. Based on the resulting 74 ore-processing plants and their energy meter's coordinates, we requested the following data from ARCOM: a) ore-processing tonnage declaration, b) type of ore benefit process and c) monthly refining and marketing of final products between 2008 and 2018. Having obtained the above-mentioned data, we proceeded to determine the energy consumption profiles for each ore-processing plant following the methodologies of Gonçalves [4] and Cabezas [1], in which the total energy consumption in comminution processes of the 74 plants was compared with the amount of ore processed over the period 2010–2018 [1,2,4].

## Declaration of Competing Interest

The authors declare that they have no known competing financial interests or personal relationships which have, or could be perceived to have, influenced the work reported in this article.
